# Comparison of Gene Expression Profile of Epiretinal Membranes Obtained from Eyes with Proliferative Vitreoretinopathy to That of Secondary Epiretinal Membranes

**DOI:** 10.1371/journal.pone.0054191

**Published:** 2013-01-23

**Authors:** Ryo Asato, Shigeo Yoshida, Atsushi Ogura, Takahito Nakama, Keijiro Ishikawa, Shintaro Nakao, Yukio Sassa, Hiroshi Enaida, Yuji Oshima, Kazuho Ikeo, Takashi Gojobori, Toshihiro Kono, Tatsuro Ishibashi

**Affiliations:** 1 Department of Ophthalmology, Graduate School of Medical Sciences, Kyushu University, Fukuoka, Japan; 2 Institute for Genome Research, The University of Tokushima, Tokushima, Japan; 3 Center for Information Biology and DNA Data Bank of Japan, National Institute of Genetics, Mishima, Japan; 4 Department of Ophthalmology, Chikushi Hospital, Chikusino-shi, Fukuoka University, Fukuoka, Japan; Massachusetts Eye & Ear Infirmary, Harvard Medical School, United States of America

## Abstract

**Background:**

Proliferative vitreoretinopathy (PVR) is a destructive complication of retinal detachment and vitreoretinal surgery which can lead to severe vision reduction by tractional retinal detachments. The purpose of this study was to determine the gene expression profile of epiretinal membranes (ERMs) associated with a PVR (PVR-ERM) and to compare it to the expression profile of less-aggressive secondary ERMs.

**Methodology/Principal Findings:**

A PCR-amplified complementary DNA (cDNA) library was constructed using the RNAs isolated from ERMs obtained during vitrectomy. The sequence from the 5′ end was obtained for randomly selected clones and used to generate expressed sequence tags (ESTs). We obtained 1116 nonredundant clusters representing individual genes expressed in PVR-ERMs, and 799 clusters representing the genes expressed in secondary ERMs. The transcriptome of the PVR-ERMs was subdivided by functional subsets of genes related to metabolism, cell adhesion, cytoskeleton, signaling, and other functions, by FatiGo analysis. The genes highly expressed in PVR-ERMs were compared to those expressed in the secondary ERMs, and these were subdivided by cell adhesion, proliferation, and other functions. Querying 10 cell adhesion-related genes against the STRING database yielded 70 possible physical relationships to other genes/proteins, which included an additional 60 genes that were not detected in the PVR-ERM library. Of these, soluble CD44 and soluble vascular cellular adhesion molecule-1 were significantly increased in the vitreous of patients with PVR.

**Conclusions/Significance:**

Our results support an earlier hypothesis that a PVR-ERM, even from genomic points of view, is an aberrant form of wound healing response. Genes preferentially expressed in PVR-ERMs may play an important role in the progression of PVR and could be served as therapeutic targets.

## Introduction

Proliferative vitreoretinopathy (PVR) is a destructive complication of retinal detachment and vitreoretinal surgeries [1]. PVR is believed to represent a maladapted retinal wound repair process with proliferation of retinal and immune cells leading to the formation of scar-like fibrous epiretinal membranes (ERMs) which can cause tractional retinal detachment (RD).

At present, surgical removal of the fibrous membranes and restoration of the physiological conditions are the first choice treatments of PVR. Although the success rates of RD surgery was significantly improved by vitrectomy combined with C3F8 gas or silicone tamponade, the surgical treatment of PVR is often unsuccessful. Therefore, the surgery needs to be supplemented by local medications to inhibit the formation of new proliferative lesions. Thus far, adjuvant treatments such as daunorubicin [2], liposomal doxorubicin [3], and a combination of 5-fluorouracil and low-molecular weight heparin [4] have been used to try to prevent the development of PVR. Unfortunately, there is no satisfactory anti-proliferative treatment available. Therefore, it is important to develop new molecular targeting therapies based on the exact pathogenesis of PVR.

The development of PVR is a complex process involving humoral and cellular factors. The results of earlier studies showed that the cells that are crucial for the formation of PVR-ERMs are retinal pigment epithelial cells, glial cells, fibroblasts, and macrophages [5]. In addition, various factors, including transforming growth factor-β2 (TGF-β2) [6], basic fibroblast growth factor (bFGF) [7], platelet-derived growth factor (PDGF) [8], tumor necrosis factor-α (TNF-α) [9], and monocyte chemotactic protein-1 (MCP-1) [10] have been shown to be involved in the pathogenesis of PVR.

Earlier conventional studies investigating the molecular factors associated with PVR have focused mainly on one or a few molecules or pathways. Therefore, a comprehensive examination of the molecular events taking place in PVR that may lead to epiretinal proliferation remains undetermined. Recent technological advancements in genomics have given investigators new opportunities to identify global gene expression in specific tissues [11]. Expressed sequence tag (EST) analysis permit the identification of genes expressed in individual tissues in a completely unambiguous manner. Thus far, several eye-related EST projects have been published utilizing whole human eyes, retinas, retinal pigment epithelial cells, ciliary body, trabecular meshwork, corneal epithelium, canine retinas, and mouse retinas [12]. However, an EST analysis has not been performed on human ERM associated with PVR (PVR-ERM) partly because of the difficulty in obtaining sufficient amounts of human ERMs.

We have succeeded in performing EST analyses of the genes expressed in epiretinal fibrovascular membranes (FVMs) from patients with proliferative diabetic retinopathy [13]–[15]. We found that unrecognized genes such as tumor endothelial cell marker 7 and periostin were highly expressed in FVMs. This indicated that a comprehensive analysis of gene expression in ERMs may be an important first step in enhancing our understanding of the formation of ERM.

Secondary ERMs form on the inner surface of the macula in eyes after intraocular surgery, e.g., after lensectomy, retinal detachment surgery, and retinal laser photocoagulation [16]. Due to the wrinkling of the retina, an ERM can cause significant distortions in the vision, i.e., metamorphopsias. However, the progression of a secondary ERM is generally less aggressive and seldom causes traction retinal detachment as do primary ERMs.

Biological events are associated with changes in the expression of crucial genes. During the onset and progression of diseases, extensive changes take place in gene expression [17]. By comparing gene expression profiles under different conditions, individual genes or group of genes that play important roles in a particular disease process can be identified.

Thus, the purpose of this study was to determine the gene expression profile of human PVR-ERMs and secondary ERMs, and to compare genes differentially expressed between PVR-ERMs and secondary ERMs. We hypothesized that this strategy would identify differentially expressed genes that might be responsible for the more aggressive behavior of PVR-ERMs. Such studies may also provide new therapeutic agents that can be targeted and thus inhibit the progression of PVR-ERMs.

## Materials and Methods

### Subjects

Procedures using human samples were conducted in accordance with the Declaration of Helsinki and approved by the Kyushu University Institutional Review Board for Clinical Research. We obtained written informed consent from all the participants.

ERMs were surgically removed from 3 eyes of patients with PVR and 2 eyes with a secondary ERM that developed after cataract surgery. The ERMs were collected during pars plana vitrectomy with membrane peeling. The ERM specimens obtained from the 3 eyes with PVR (PVR-ERM; 62, 66, and 66 years) were processed for cDNA library construction. We also collected vitreous samples from 11 eyes of 11 patients (age, 59.3±9.4 years; men∶women, 7∶4) with PVR during pars plana vitrectomy. For control, vitreous samples were collected from 26 eyes of 26 patients (age 69.8±10.2 years; men∶women, 11∶15) who were undergoing ERM surgery.

### RNA Extraction

All of the resected tissues were snap frozen and stored at −80°C. To prepare total RNA, the tissue was homogenized with a MagNA Lyser Green Beads kit (Roche Applied Science, Mannheim, Germany) according to the manufacturer's instructions. Total RNA was extracted with TRizol (Qiagen, Germantown, MD) and exposed to DNase (RNase-free DNase set, Qiagen) to eliminate potential genomic DNA contamination.

### cDNA Synthesis

To overcome the limitation of the starting amounts of RNA, SMART™ (Switching Mechanism at the 5′ end of RNA Transcript) technology, an exponential PCR-based method, was employed as described in detail [18]. Briefly, the total RNA was reverse-transcribed using the SMART cDNA Library Construction Kit (Clontech, Palo Alto, CA) according to the manufacturer's protocol. Annealing was done at 70°C for 2 minutes in the presence of the SMART oligosequence; (AAGCAGTGGTATCAACGCAGAGTGGCCATTACGGCCGGG), and the overhang of the oligo(dT) primer (ATTCTAGAGGCCGAGGCGGCCGACATG [dT]30VN). The reaction was followed by the addition of Superscript reverse transcriptase (RT; Gibco-BRL, Gaithersburg, MD), and the mixture was incubated at 37°C for 1 hour (final volume, 20 µl).

Representative double-stranded cDNAs were then generated by exponential PCR amplification. Two microliters of each cDNA fraction was amplified in a 100 µl reaction containing a final concentration of 1× PCR reaction buffer (Advantage 2 PCR kit (BD Biosciences Clontech)), 0.2 mM dNTPs, 0.5 pmol/ µl forward primer (AAGCAGTGGTATCAACGCAGAGT), 0.5 pmol/ µl reverse primer (ATTCTAGAGGCCGAGGCGGCCGACATG), and 10 U/ µl Advantage 2 enzyme mix (Clontech). The number of cycles needed for exponential phase amplification of this cDNA was determined by running a series of 10 µl analytical PCR amplifications at 18, 20, and 22 cycles using the same kit. We chose 20 cycles for the library construction.

### Size Fractionation of cDNA and Library Construction

To prevent an over representation of clones with short inserts, the resultant cDNAs were size-fractionated using agarose gel electrophoresis. For this, a PCR-amplified sample was loaded into a single well of a 2% low-melting agarose gel. Three separate slices corresponding to approximate molecular weights of 0.5 to 1 kb, 1 to 2 kb, and more than 2 kb were cut from the gel and melted at 65°C for 10 minutes [18]. cDNA was extracted from the gel slices with agarase (Gelase, Epicentre, Madison, WI) according to the manufacturer's instructions. One-tenth of each eluted cDNA was used for ligation into a cloning vector, pCR4-TOPO (Invitrogen, Carlsbad, CA), according to the manufacturer's protocol [19]. This was followed by transformation of *Escherichia coli* competent cells (MAX Efficiency DH5α Chemically Competent Cells (Invitrogen) and plated onto ampicillin-agar plates.

### Sequence Analysis and Functional Annotation

Approximately the same number of colonies was randomly selected for DNA sequencing. The colonies were inoculated into individual wells of 96-well plates containing 150 µL of LB media, and incubated at 37°C for 18–22 h. Frozen glycerol stocks were prepared by adding 75 µL of 50% glycerol to each well, and the plates were stored at −80°C. Double-stranded cDNAs were obtained for sequencing either by miniprep (Invitrogen) or by PCR amplification directly from frozen glycerol stocks as described [20]. DNA sequencing from the 5′ end of the cDNA insert was carried out using SMART 5′ PCR primer with an automated sequencer (Applied Biosystems Inc, Foster City, CA, USA) using standard protocols [21]. Data were analyzed with PHRED [22] to identify and trim quality reads. Human mitochondrial sequences were trimmed or eliminated using Cross-match programs. All sequences have been deposited in DNA Data Bank of Japan (accession numbers: HX784107–HX787324).

EST sequences were assembled and clustered by the method used by Ogura et al. [23] in which the gene-clustering method, BLASTCLUST, was performed to obtain a rough estimation of clusters containing similar sequences. The Phragment Assembly Program (PHRAP) was used to assemble the sequence in the estimated clusters. Functional annotation was conducted on the nonredundant data set of human ERM ESTs. Gene ontology (FatiGO) was used to categorize human eye ESTs with respect to Kyoto Encyclopedia of Genes and Genomes (KEGG) biological pathways [24].

To identify library-specific genes that were differentially expressed in PVR-ERM and secondary ERM, we used the method described in Susko and Roger [25] based on Binomial and Chi-square tests for the expression frequencies patterns in which genes occur in each library.

### Enzyme-linked immunosorbent assay (ELISA) for sCD44 and sVCAM-1

The levels of both CD44 and VCAM1 in the vitreous fluid from the same eye were measured with an enzyme-linked immunosorbent assay (ELISA) for human sCD44 (Gen-Probe, San Diego, USA) and sVCAM-1 (RayBiotech; Norcross, GA). Each assay was performed according to the manufacturer's protocols as explained in detail in our publication [26]. The levels of sCD44 and sVCAM-1 in the vitreous fluid were within the detection range of the respective assays; the minimum detectable concentration was 0.113 ng/mL for sCD44 and 0.3 ng/mL for sVCAM-1. The intra-assay coefficient of variation [CV] was 1.52% and the inter-assay CV was 3.08% for sCD44, 10% and 12% for sVCAM-1.

### Statistical Analyses

Statistical analyses were performed using JMP (version 7.0, SAS Institute, Cary, NC), a commercial statistical software package. The distribution of the data was first determined by the Shapiro-Wilk tests. The significance of the differences in the sCD44 and sVCAM-1 levels between the different groups was determined with the Mann-Whitney test. The correlation between the levels of sCD44 and sVCAM-1 was determined by the Spearman coefficient of correlation.

## Results

### Construction of human ERM cDNA libraries and EST analysis

We constructed a cDNA library from resected PVR-ERMs and secondary ERMs with SMART technology. Agarose gel electrophoresis of amplified PCR isolates had a smear band that extended from 0.1 kb to >4 kb (data not shown). We performed 2688 single-pass sequence analysis from the 5′end of the individual clones from the PVR-ERMs and on 1632 secondary ERMs cDNA library (Table 1). Of these 2688 and 1632 clones, 323 and 237 clones, respectively, were of low quality or were repetitive sequences and were deleted from further analysis. Through the EST assembly system, 2365 high-quality ESTs from PVR-ERMs were clustered 1395 high-quality ESTs from secondary ERMs were clustered and assembled to 799 nonredundant clusters representing individual genes.

**Figure 1:**
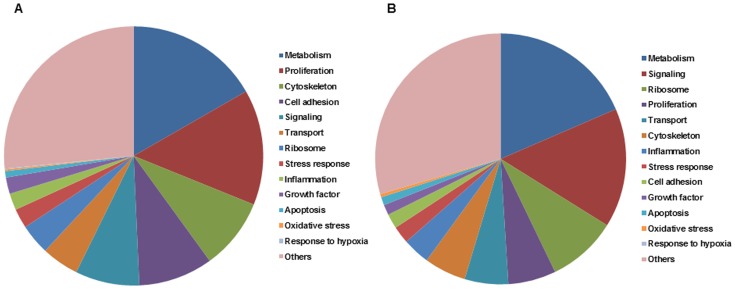
Known human genes identified in the human epiretinal membrane (ERM) associated with PVR (A) and secondary ERM (B) are grouped according to the KEGG functional categories.

We next performed sequence similarity searches to compare every EST to those in public databases. For ESTs with known gene matches in public databases, functional annotations were retrieved from the Ensembl database and analyzed by FatiGo. Among the 1116 nonredundant cluster derived from the PVR-ERM library, 916 (82%) matched the human cDNA database (Ensembl). The remaining 200 (18%) corresponded to potentially new ESTs or untranslated sequences. Among the database-matched, 916 clusters were subdivided to functional subsets of genes related to metabolism, cell adhesion, cytoskeleton, signaling, and other functions by FatiGo analysis (Figure 1A). Among these, metastasis associated lung adenocarcinoma transcript 1 (*MALAT1*) and fibronectin precursor (*FN1*) appeared to be the most abundant transcripts in the PVR-ERMs (Table 2). All non-redundant sets of ESTs expressed in PVR-ERMs are shown in the Table S1.

Among the 799 nonredundant clusters derived from the secondary ERM libraries, 637 (80%) matched the human cDNA database (Ensembl). The remaining 162 (20%) corresponded to new ESTs or untranslated sequences. Among the database-matched 799 clusters, 637 were subdivided by functional subsets of genes related to metabolism, signaling, ribosome, cytoskeleton, and other functions by FatiGo analysis (Figure 1B). Among these, Zinc finger protein 713 (*ZNF713*) and forkhead box K1 (*FOXK1*) were the most abundant transcripts in the secondary ERM (Table 3). All non-redundant sets of ESTs expressed in secondary ERMs are shown in the Table S2. Approximately one-third of the ESTs were common to PVR-ERMs and secondary ERMs. All sequences have been deposited in DNA Data Bank of Japan.

We then determined the genes that were differentially expressed in the PVR-ERMs and secondary ERMs using the approaches proposed by Susko and Roger [25]. Based on their methods, four genes, *MALAT1, ZNF713, FN1*, and *PARP8*,were abundantly represented (*P*-value<B-H cutoff) in the two libraries were identified. The data also showed that 52 genes were highly represented in either PVR-ERMs or secondary ERMs (*P*-value<0.1). Twenty-three genes were expressed at higher levels in the PVR-ERMs and 29 genes were expressed at higher levels in secondary ERMs (Table 4).

The genes highly expressed in PVR-ERMs were subdivided by functional subsets of those related to cell adhesion, proliferation, and other functions. In contrast, the genes related to ribosomes, metabolism, and signaling were preferentially up-regulated in secondary ERMs (Table 4).

### *In Silico* gene/protein network analysis

To identify potential biological relationships among the genes expressed in the ERMs, we used the recently developed Search Tool for the Retrieval of Interacting Genes (STRING) 9.0 database (http://string-db.org). STRING is a web-based software that can extract protein-protein interactions that include direct (physical) and indirect (functional) associations [27]. We queried the gene symbols of 916 and 637 genes from the PVR-ERM and secondary ERM cDNA libraries, respectively, against STRING and obtained predicted interactions for genes/proteins. Of the 916 genes submitted to the PVR-ERM cDNA library, 843 (92.0%) yielded 4274 possible physical relationships to other genes/proteins which included 3358 proteins that had not been detected in the PVR-ERM library (Table S3).

Of the 637 genes submitted to the secondary ERM cDNA library, 561 (88.0%) yielded 3270 possible physical relationships to other genes/proteins which included 2633 proteins that were not detected in the secondary ERM library (Table S4).

**Figure 2:**
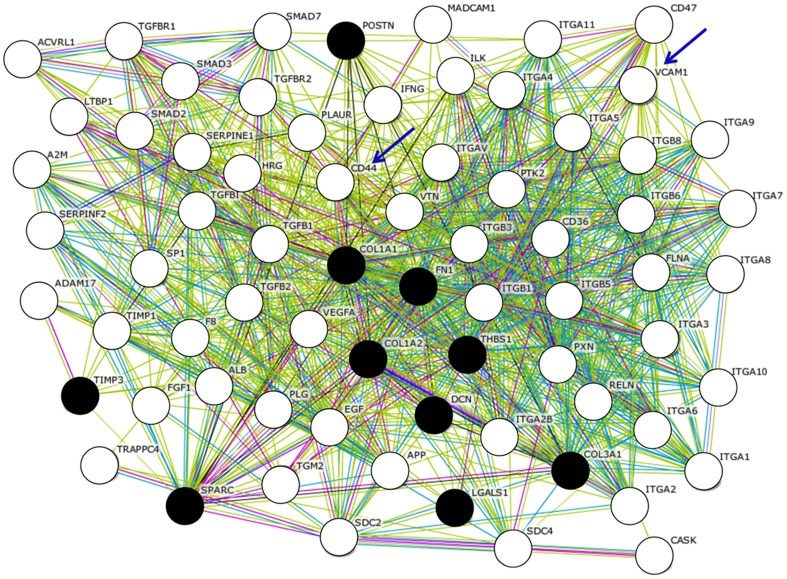
Molecular networks associated with the genes expressed in ERMs associated with PVR (PVR-ERM) are shown. Gene symbols of 10 cell adhesion-related genes (*FN1, COL1A2, COL1A1, COL3A1, TIMP3, LGALS1, THBS1, DCN, POSTN, SPARC)* from the PVR-ERM cDNA library were queried against the STRING database, and the predicted interactions for genes/proteins were obtained. Filled black circles represent the submitted 10 genes/proteins from the PVR-ERM cDNA library, and the white circles represent potentially expressed 60 genes in PVR-ERMs that are extracted *in Silico*. Of these, CD44 and VCAM-1 were examine by ELISA and are shown by arrows. The gene names are shown next to the circles. The edges connecting two circles represent the predicted functional associations. An edge is drawn with up to 7 differently colored lines. These lines represent the presence of the seven types of evidence used in predicting the associations. A red line indicates the presence of fusion evidence; a green line-neighborhood evidence; a blue line–co-occurrence evidence; a purple line-experimental evidence; a yellow line-textmining evidence; a light blue line-database evidence; and a black line–co-expression evidence.

Genes related to cell adhesion are supposed to be characteristic components of PVR-ERMs (Table 4). The gene symbols of 10 cell adhesion-related genes, *FN1, COL1A2, COL1A1, COL3A1, TIMP3, LGALS1, THBS1, DCN, POSTN, SPARC*, that were preferentially expressed in the PVR-ERMs cDNA library, were queried against the STRING database. This yielded 70 possible physical relationships to other genes/proteins, which included an additional 60 genes that were not detected in the PVR-ERM library (Figure 2).

### Enzyme-linked immunosorbent assay (ELISA) for *In Silico* Extracted Proteins

**Figure 3:**
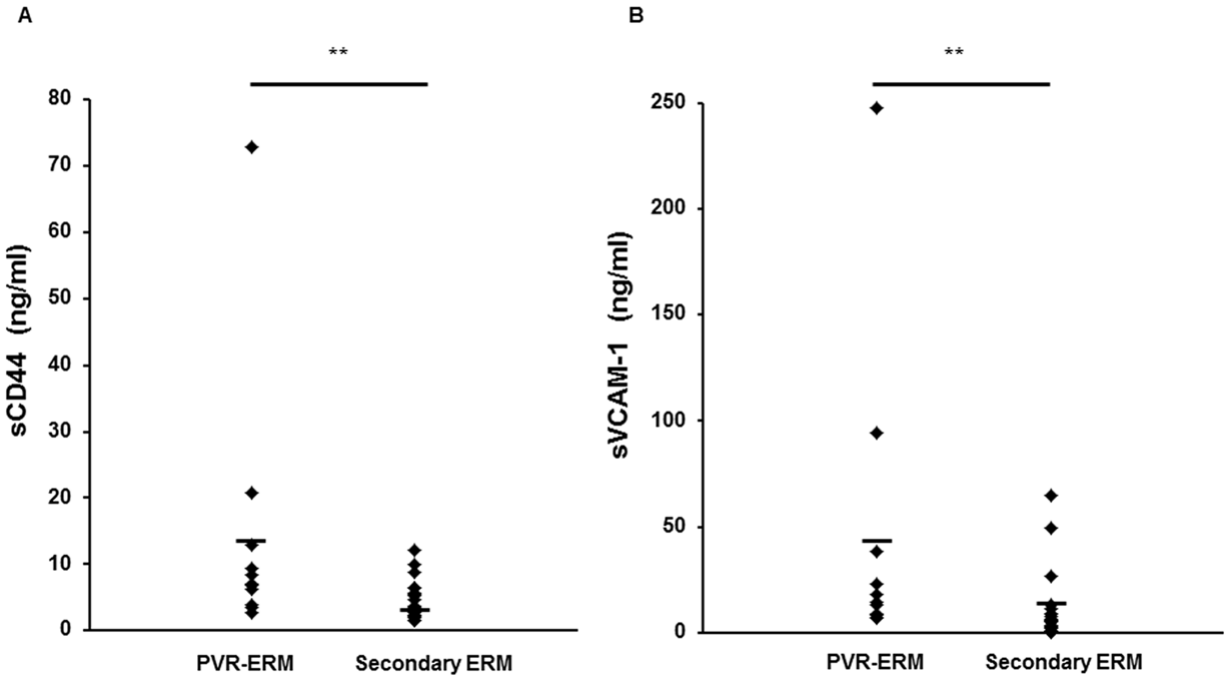
sCD44 (A) and sVCAM-1 (B) concentrations in the vitreous fluid of patients with secondary ERM and proliferative vitreoretinopathy patients (PVR-ERM). The levels of both sCD44 and sVCAM-1 were significantly higher in the patients with PVR than in the eyes with secondary ERM (*P<0.001).

To determine if these possibly related genes/proteins extracted only from the public database *in silico* are indeed elevated in the vitreous of patients with PVR, we chose CD44 and VCAM-1 (Figure 2, arrows) which were extracted as interacted nodes by molecular network involved in cell adhesion-related genes detected in the PVR-ERM library. We examined the concentration of these two molecules, in 11 vitreous samples of patients with PVR collected during vitrectomy, and in the 26 vitreous samples obtained from patients with secondary ERM. The concentration of both sCD44 and sVCAM-1 in the vitreous was significantly higher in the patients with PVR (14.05 ng/mL, range, 6.89–72.87 ng/mL; and 43.50 ng/mL, range, 8.04–247.35 ng/mL), than in eyes with secondary ERM (4.26 ng/mL, range, 1.47–12.06 ng/mL; and 9.15 ng/mL, range, 0–64.70 ng/mL; *P*<0.*01*; Figure 3).

**Figure 4:**
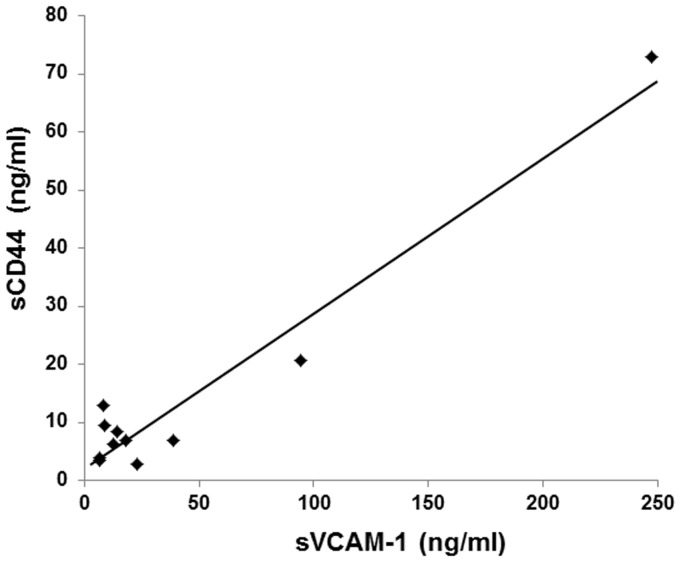
Correlations of vitreous sCD44 and sVCAM-1 levels in patients with PVR. There was a strong statistically significant correlation between the vitreous concentration of sCD44 and sVCAM-1 (*r* = 0.971;*P*<0.0001).

The correlation between the vitreous concentrations of sCD44 and sVCAM-1 was statistically significant (r = 0.971; *P*<0.0001; Spearman correlation coefficient; Figure 4).

## Discussion

We searched Medline with key words “epiretinal membrane” and “gene expression profiling”, and did not find any papers reporting a profile of the gene expression in human PVR-ERMs or in secondary ERMs. The EST classification by BLAST suggested that the ERMs, especially those associated with a PVR, result from a complex pathological process with alterations in the expression of a variety of functional genes. These genes play important roles in the progression of PVR-ERMs.

The most highly expressed gene in the PVR-ERM was *MALAT1*, a long, non-coding RNA that regulates the processing pre-mRNAs in mammalian cells. It is associated with metastasis, and it regulates cell motility through a concomitant regulation of the expression of motility-related genes by transcriptional and/or post-transcriptional regulation [28]. However, *MALAT1* has not been shown to be synthesized by PVR-ERMs. The strong expression of *MALAT1* in PVR-ERMs could be explained by the need for extensive migration of PVR-ERMs on the retina.

The PVR process resembles an aberrant wound-healing response in which several stages can be distinguished: attachment, migration, and proliferation of cells; deposition and remodeling of the extracellular matrix; and contraction [29]–[32]. Comparisons of the gene expression profile of PVR-ERM to that of secondary ERMs showed an increased expression of genes involved in cell adhesion and proliferation in the PVR-ERMs (Table 4). This is consistent with an aberrant wound-healing response.

Among the components of the extracellular matrix, type II collagen, secreted protein acidic, cysteine-rich (SPARC), thrombospondin (THBS), and fibronectin (FN), are also major components of the vitreous [33], [34]. Indeed, FN is the second most abundant transcript in the PVR-ERM library (Table 2). However, our EST analyses demonstrated an increased expression of *FN1, COL1A2, COL1A1, COL3A1, TIMP3, LGALS1, THBS1, DCN, POSTN, SPARC* as cellular adhesion components (Table 4). This suggests that cells that comprise the PVR-ERMs actively produce a variety of cell adhesion-related molecules and act in an autocrine fashion. These results are in good agreement with a morphological study showing that the amount of extracellular matrix in ERMs was positively correlated with the disease process, i.e., greater in PVR than slowly progressive ERM [35].

We have recently demonstrated that periostin, a secreted extracellular matrix (ECM) protein that is found in areas of pathological fibrosis, may play significant roles in the development of fibrovascular membranes (FVMs) associated with PDR [14]. The results of the present study showed that the *POSTN* is also highly expressed in PVR-ERMs, and the periostin protein is markedly increased in the vitreous of patients with PVR (Ishikawa K and Yoshida S, manuscript in preparation) as is PDR. These observations strongly support the idea that periostin is closely involved in the proliferation of ERMs commonly associated with both PDR and PVR. Whether periostin might serve as new molecular target to inhibit epiretinal fibrous proliferation awaits further studies.

Several genes related to proliferation, viz., *MALAT1, SERPINE1, CD320*, and *STAT3*, were up-regulated in PVR-ERMs. These findings are in agreement with those obtained from histological studies showing rapidly growing PVR-ERMs had the highest density of cells and the largest number of anti-Ki-67 labeled cells [36]–[38]. Thus, proliferation is most likely a major contributor to the rapid expansion of PVR-ERMs.

In comparison to the genes preferentially expressed in PVR-ERM, genes preferentially expressed in the secondary ERMs were related to ribosome and metabolism and might serve a housekeeping role (Table 4). This suggests that many of the actively expressed genes are housekeeping genes in secondary ERMs, and the tissue is relatively more resting in comparison to PVR-ERMs. This then indicates that the aforementioned genes that are preferentially increased in PVR-ERM may represent an aggressive phenotype of PVR thus may be attractive therapeutic targets to inhibit the progression of PVR.

Although our approach obtained important information, there is still one step that requires further study. Our current EST study lacks depth of sequencing. To reinforce the relatively low representation, we attempted to use a bioinformatic approach with the STRING software [27]. Because analyzing tens of thousands sequences is time-consuming and costly, we used *in Silico* analyses which effectively uses the wealth of public databases to develop a more comprehensive gene expression signature associated with PVR-ERMs. Although the database is biased toward well-studied genes relative to newly discovered genes, it offers a method for rapidly establishing potential associations between genes and functional pathways (Figure 2). In support of this, we were able to demonstrate that CD44 and VCAM-1, which were extracted only from the public database *in Silico* are indeed elevated in the vitreous of patients with PVR, and that there is a strong correlation between the two molecules in the development of ERMs (Figures 3 and 4). This is in parallel with our previous study that showed that more than 90% of computationally extracted biologically-related candidate genes were confirmed to be expressed in FVMs [13], and may intensify the usefulness of the bioinformatic approach to further extract important genes.

CD44, a cell-surface adhesion molecule and receptor for hyaluronan, plays an important role in cell migration and tumor growth and progression [39]. Additionally, it was recently shown to be involved in the TNF-α-induced epithelial-mesenchymal transition [40]. VCAM-1 may play a pathophysiologic role both in immune responses and in leukocyte emigration to sites of inflammation through interaction with VLA4 [41]. In spite of the presumed functions of these two molecules, both sCD44 and sVCAM-1 may play significant roles in the development of PVR, but no direct evidence has yet been reported. Moreover, the up-regulation and strong correlation of sCD44 and sVCAM-1 found in the vitreous of patients with PVR indicates that these two molecules are involved in the progression of PVR in a coordinated manner. Therefore, further studies are required to determine the role played by sCD44 and sVCAM-1 in the pathogenesis of PVR.

In summary, we have generated a profile of the gene expression in human ERMs. Our study supports the previous hypothesis that formation of PVR-ERMs, even from genomic points of view, is an aberrant form of healing response. This consists of cellular proliferation, migration, extracellular deposition, and contraction. In combination with the bioinformatic approach, we were able to obtain new evidence that such molecules as periostin and CD44 are possibly involved in the molecular pathways associated with the formation of PVR. Further investigations of these newly detected genes are needed to determine whether they can be new targets for combating the development and progression of PVR-ERMs.

## Supporting Information

Table S1 **The most abundantly represented genes in the PVR-ERMs cDNA library.** The bold letter shows that the genes were expressed both the PVR-ERMs and Secondary ERMs.
(XLSX)

Table S2 **The most abundantly represented genes in the Secondary ERMs cDNA library.** The bold letter shows that the genes were expressed both the PVR-ERMs and Secondary ERMs.
(XLSX)

Table S3 **Possible physical interactors to the proteins encoded by cDNAs expressed in the PVR-ERMs library.**
(XLSX)

Table S4 **Possible physical interactors to the proteins encoded by cDNAs expressed in the Secondary ERMs library.**
(XLSX)
