# Correction: Comparison of Gene Expression Profile of Epiretinal Membranes Obtained from Eyes with Proliferative Vitreoretinopathy to That of Secondary Epiretinal Membranes

**DOI:** 10.1371/annotation/6032dd20-65b4-4d4d-bef6-7ba2ef6d3591

**Published:** 2013-04-23

**Authors:** Ryo Asato, Shigeo Yoshida, Atsushi Ogura, Takahito Nakama, Keijiro Ishikawa, Shintaro Nakao, Yukio Sassa, Hiroshi Enaida, Yuji Oshima, Kazuho Ikeo, Takashi Gojobori, Toshihiro Kono, Tatsuro Ishibashi

Tables 1-4 are missing from the publication. They can be viewed here:

Table 1: 

**Figure:**
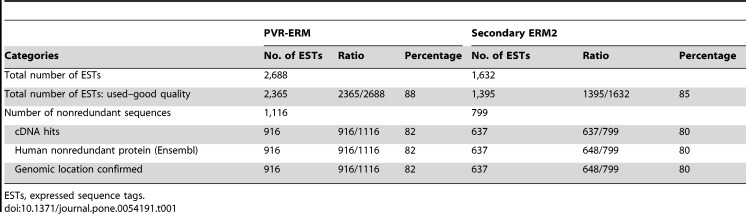


[^]

Table 2: 

**Figure:**
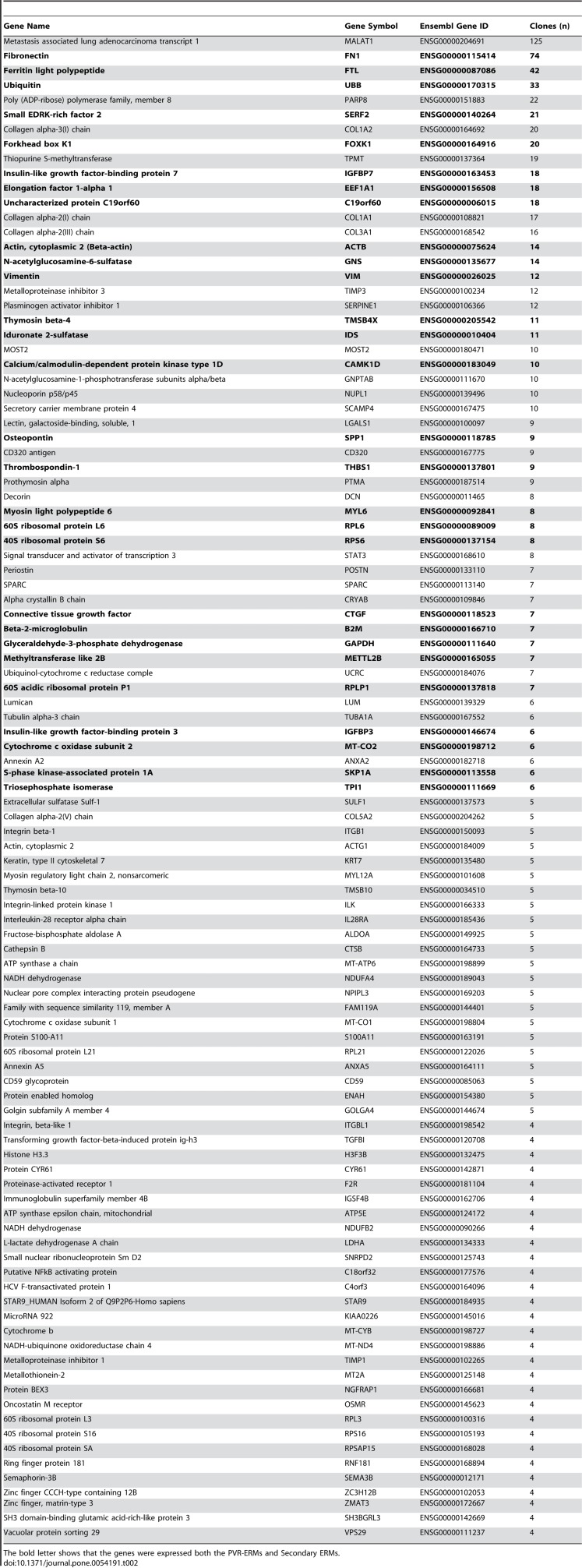


[^]

Table 3: 

**Figure:**
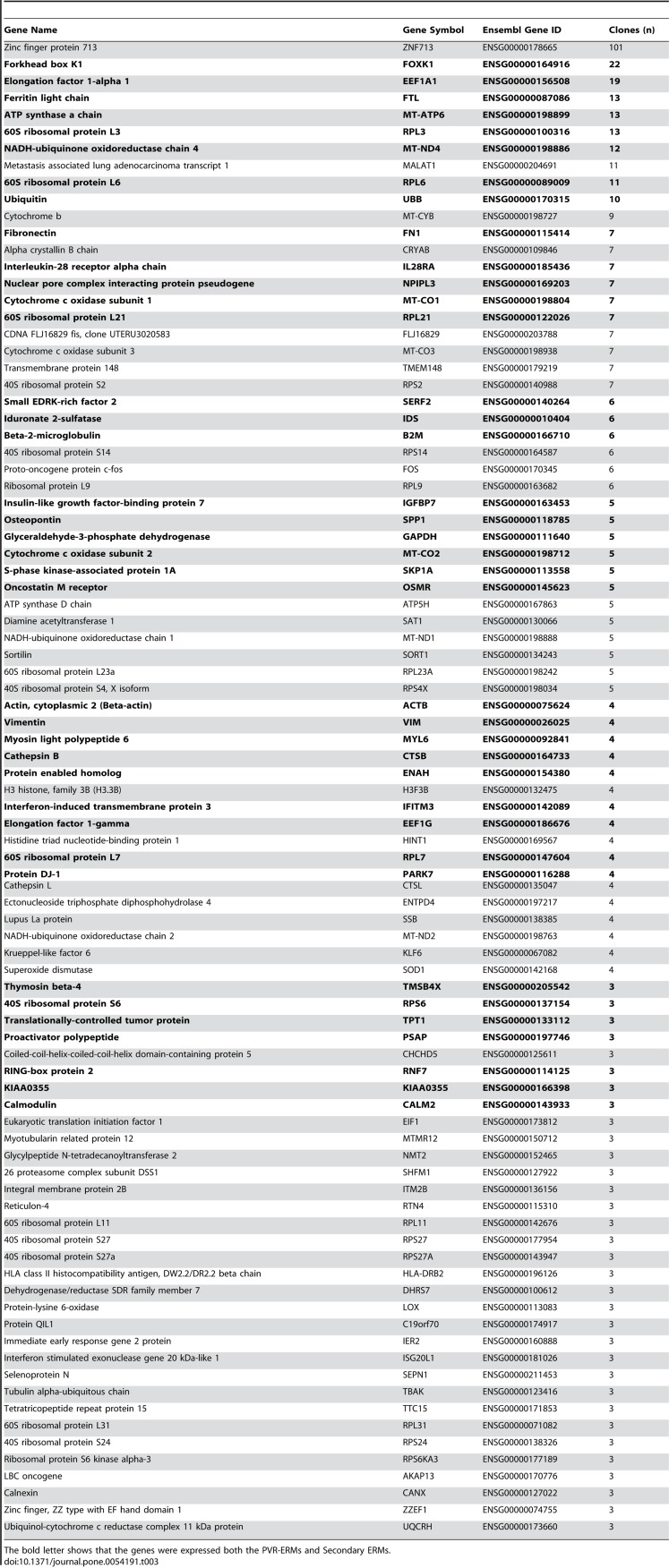


[^]

Table 4: 

**Figure:**
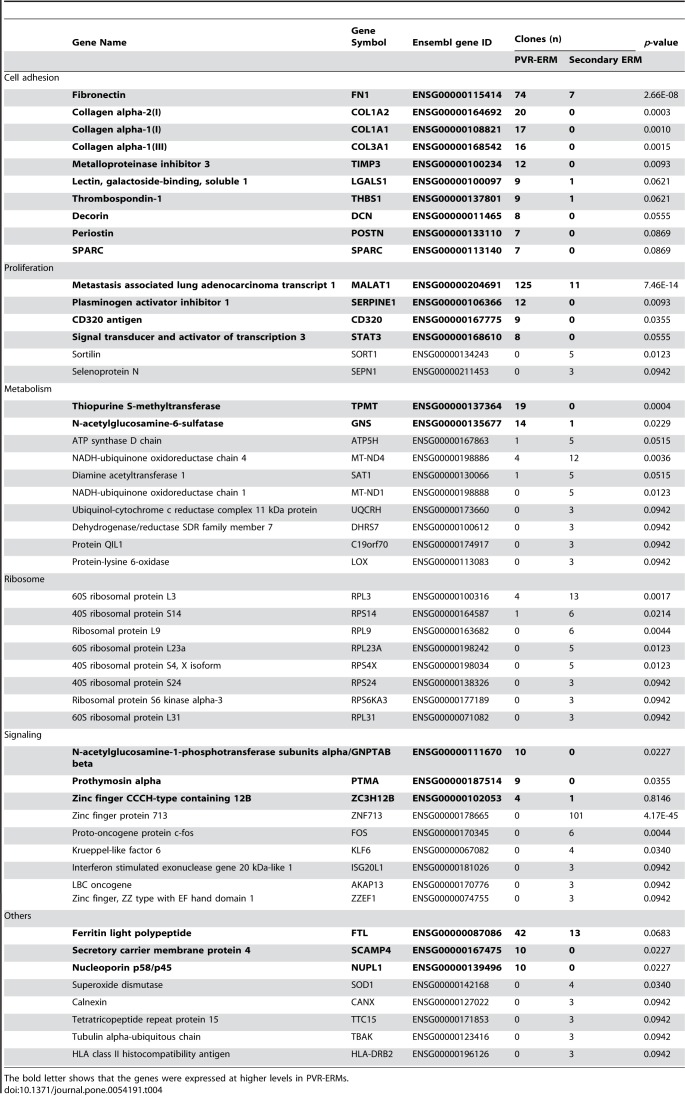


[^]

Affiliation 4 is incorrect. The correct affiliation is:

Department of Ophthalmology, Fukuoka University Chikushi Hospital, Chikusino-shi, Fukuoka, Japan.

There was an error in the funding information. The correct funding statement is:

Supported in part by grants from the Ministry of Education, Science, Sports and Culture, Japan (TI and SY), and Takeda Science Foundation (SY). The funders had no role in study design, data collection and analysis, decision to publish, or preparation of the manuscript. 

